# Foreign Summary

**Published:** 1838-03-28

**Authors:** 


### FOREIGN SUMMARY.

*On the efficacy of Sugar of Lead in cases of Fever with Ulcers of the Intestines.*

The treatment recommended in the following article has recently acquired a fresh interest from the recommendation of the same remedy in the Asiatic Cholera.

A fever has prevailed for some time at Bonn, attended by a peculiar appearance of the tongue; pain in the abdomen, especially on the right side, upon pressure: nervous symptoms, and a particular state of the pulse; it is also accompanied by diarrhœa, and, on examining those who had died of it, the termination of the small and the commencement of the large intestines have been found in a degenerated state. During the inflammatory stage of the disease, Dr. Nasse applied cupping glasses to the abdomen, and in most cases more than once; in a few cases, when the pulse was hard, he bled from the arm, and in every case applied blisters to the abdomen. As soon as the diarrhœa made its appearance, sugar of lead was prescribed, generally without being combined with opium, and was continued until the fœces re-assumed their natural appearance, and until the abdomen ceased to be pained by pressure. Dr. L. Jung, in his work *De Dothienenteritide ejusque plumbo acetico sanandi ratione*, had already described the admirable operation of this medicine. Dr. Nasse gives a similar testimony to its value, and states that, in his own practice, it has, in his opinion, saved several lives, which would have been lost without it. Nineteen cases of the above fever were treated with sugar of lead; five in children, and fourteen in adults; of these eighteen recovered. The lead was not administered till the fifth, eighth, or tenth day of the complaint, or even later, when cerebral congestion rendered necessary the application of leeches. The dose was one-fourth, one-third, and half a grain, from three to six times daily. Generally, two grains were sufficient to put a stop to the diarrhœa; but, in five cases, four grains were necessary. One of the patients, who had forty stools in twenty-four hours, and who suffered from tenesmus, took in all eight grains, at first without, and afterwards combined with opium, and at last with starch clysters. The young man who died, took altogether ten grains; in his case, the diarrhœa yielded to it in a great degree, but the abdominal irritability and the tympanitis were such as to induce its fatal termination. As soon as further doses of lead seemed unnecessary, a weak infusion of ipecacuanha was given. In some of the cases which recovered there had been petechiæ; and in some, too, the evacuations had been bloody; but the lead had been administered all the same. Opium was not generally ordered, on account of its effect upon the head. In eight cases, on account of weakness and fainting fits, the liq. ammon. carb. was had recourse to; in one, that of a child, quinine was given before the lead, but, the fever increasing, and blood passing off with the stools, the latter was prescribed, and with the best effect.—*Brit. and For. Med. Review, from Pr. Med. Zeitung.*

*On the external use of Calomel in Inflammation of the Eyes, by Dr. Fricke, of Hamburg.*

The author calls attention to a remedy, now fallen much into disuse, in cases of ophthalmia. He has employed it extensively, and the success with which its application has been attended, makes him anxious to render it better known generally. The first cases which he selected for its use, were of a chronic character, and such as had been subjected, without advantage, to other treatment; e. g. rheumatic catarrhal, and scrofulous inflammation, attended with the various changes which frequently accompany them. Subsequently he employed the remedy in other and milder forms of inflammation; the result of his observations being, that calomel, employed in this manner, is of the greatest utility. In cases of rheumatic inflammation of the eyes, in which, notwithstanding the most rational treatment, local and general means, derivants, narcotics, antirheumatics, &c., the redness, pain, and intolerance of light have continued, the calomel in the hands of the author, has been attended, in a couple of days, or even after a single application, with an amelioration, or even a disappearance, of all these symptoms. Instead of the great intolerance of light, the eye becomes capable of bearing its stimulus; the burning hot tears become milder and less abundant; the sensation of pain in the eye gradually diminishes, and the patient is soon cured.

The same pleasing results follow the use of calomel in strumous ophthalmia. In the management of that most distressing symptom of this disease, intolerance of light, the author is not aware of one case in which the calomel was applied to the eye, where its application was not followed by success.

In other inflammations of the eyes, in which a general plan of treatment had failed, and in which the calomel was employed experimentally, to observe its action, two only are known to Dr. Fricke, in which any injurious effects were produced.

The mode of employing the calomel is as follows:—Dip a miniature pencil into calomel washed in alcohol, and apply it immediately to the eyeball. This application should be repeated once or oftener in the twenty-four hours. The sensations produced in the eye are very different; in a healthy eye, in general, scarcely any sensation is produced, or at most a transient and slight warmth; in inflamed eyes, pain is produced, but it is generally inconsiderable, and only complained of in certain cases. The pain disappears wholly in from half an hour to two hours. One patient felt no pain, but said that the calomel produced a feeling of coldness. Others, to whom Dr. Fricke has communicated the results of his own experience with the use of calomel, have found them in their own practice confirmed.—*Brit. and For. Med. Review, from Zeitschrift für die gesammte Medicin.*

*On the Infusion of Digitalis in Delirium Tremens.*

The infusion, prepared with a drachm of the leaves to a pint of water, was administered by Dr. Magnus Huss, of Stockholm, in six cases, all of them men of strong constitution, of from twenty-four to thirty-three years of age. Two of the cases required bloodletting. In three, a tablespoonful of the infusion was administered every hour throughout the day only; in the other, a like quantity was exhibited every hour both day and night. In the former number, the disease yielded, and sleep ensued on the third day; in the latter number, sleep ensued in thirty-six hours; an equal quantity of the infusion was thus required in both cases. The patients awoke after a sleep of from six to ten hours, free from the disease, but labouring more or less under the effects of the medicine. In one patient the pulse had sunk to thirty-five beats, but in the other the rhythm was normal; in the whole number the pupils were contracted, and they complained of dryness of the mouth, burning in the throat, humming in the ears, heaviness in the head, great weakness, and nausea; which last symptom was so severe in one patient, that for two days he vomited whatever he took.—*Brit. and For. Med. Review, from Jahrbücher der Gesammten Med.*

*History of a Cæsarean operation practised successively, on the 20th December,* 1834, *both in respect to the mother and the child, by* Dr. Stoltz, *Professor to the Medical Faculty of Strasbourg.*

The subject of this case was a Jew girl of Strasbourg, 26 years of age, who had been very ricketty in early life. She was only 3 feet 8 inches in height, and the dimensions of the pelvis made natural exclusion of a full grown fœtus altogether impossible.

The operation was commenced with an incision made by a convex bistoury, having its cutting edge of convex form. It began two inches and a half above the *symphysis pubis*, and extended along the *linea alba*, two inches and a half above the umbilicus, keeping a little to the left of the latter. The author then cut cautiously through the skin and cellular membrane, so as to expose the aponeurosis and peritonæum, which he raised with the dissecting forceps and entered after the manner of a herniary sac, when some drops of serum came from the abdominal cavity. He then changed his convex cutting bistoury for a concave one with a button at its end, and with this he cut through the aponeurosis and peritonæum, first downwards, and then upwards, the whole extent of the external incision. The wound was then enlarged at its extremities, so as to make its whole length about seven inches. The uterus was by this means exposed, and the lips of the wound became considerably separated. A

portion of intestine presented itself, which was readily put aside. The uterus having been ascertained to be in its proper position, a careful dissection was made through its coats by means of the convex bistoury. The uterine fibres gradually retracted during the division, and the last or innermost layer gave way of itself. The whole thickness of the parietes of the uterus was from four to five lines; and when the division was complete, the membranes of the ovum exhibited themselves. The opening into the uterus was enlarged with the curved and armed bistoury. Before rupturing the membranes, care was taken to keep the parietes of the abdomen so close to the uterus as to prevent the escape of the *liquor amnii* into the abdomen. The membranes were opened with a bistoury, when three or four ounces of water escaped; and the right side of the back of the fœtus exhibited itself. With his left hand, the author took hold of the feet, which he got out without difficulty, and then readily extracted the trunk and head, using the same measures with the arms as in an ordinary footling case. The child, a female, then began to cry. It was full-grown; was eighteen inches in length, and weighed five pounds and three-quarters. The cord was tied and cut in the usual way.

The void produced by the contractions of the uterus made it very important to take every precaution to prevent the protrusion of the intestines. It was necessary likewise to check the immediate discharge of the remainder of the *liquor amnii*, and of the effused blood. Dr. Stoltz waited some minutes for a sufficient contraction of the uterus, and then he withdrew the placenta, which was attached to the posterior surface of the uterus, a little to the right. Soon afterwards, though with more difficulty, he succeeded in extracting the membranes. The uterus now retired within the pelvis, when a portion of the small intestines appeared in the lower part of the wound and a portion of omentum in the upper. The former was readily reduced, but on pushing the latter, a hiccup took place, which, however, soon went off, on the careful replacement of the parts concerned. The lips of the wound were then brought together carefully, and four ligatures applied at equal distances from each; a seton being introduced into the uterus, to facilitate the escape of secreting fluids. The operation lasted half an hour, and was borne heroically. There was little hemorhage. The patient, during the operation, was placed horizontally on her back, with her legs slightly bent, and knees brought together; care being taken to make prudent pressure laterally and from beneath.

The whole process of treatment extended to seventy days from the operation, before the patient could be said to be quite recovered from its effects. The mother and child were both in good health on the 15th September, 1835.

*Memoires de l'Academie Royale de Medicine, Tome V.* 1836. *Ed. Med, and Surg. Journal, Jan.* 1838,

---

*Speculum Uteri made of Glass.* Dr. Hacker, of Leipzig, uses a speculum in examining the vagina and womb. It consists of a simple oval cylinder, the glass of which is about two lines in thickness, its length being from four to six inches. The diameter of the end which is introduced into the vagina is half or three-quarters of an inch by a quarter or half an inch. This extremity is furnished with a blunt, rounded, and tumid border, whilst the cylinder at the other end is funnel shaped. Its form corresponds essentially with that of other specula, except that its circumference is less. The advantages of these specula of glass are their cheapness, and the facility with which they can be cleansed. When, too, they are introduced into the vagina, its mucous membrane—immediately before the tumid extremity of the speculum—can well be examined. If the material be transparent glass, by means of a bright artificial light, the parts of the vagina which are covered by the speculum may be examined. Specula of dark glass are better fitted for examining the mouth of the womb.—*B. and F. Med. Review, from Jahrbücher der in-und-ausländischen Medicin.*

---

*Vagitus Uterinus before the Rupture of the Membranes.*—As Dr. Dressel was sitting one evening with his wife, aged thirty-one, who was pregnant for the third time, he heard two evident cries from the child in the mother's womb. At the same moment his wife looked at him alarmed, placed her hand upon her right side, and declared that the cry, which she also had heard, had proceeded from that spot. The doctor immediately applied his hand to the part, and felt the child move. In the moment of the cry the child had moved violently, and had caused a perceptible pressure on the right side of the scrobiculus cordis. The mother had been healthy throughout the whole pregnancy; there were no signs of delivery, which first commenced forty-eight hours afterwards, and ended by the birth of a fine girl. This case Dr. D. relates as a new instance of the possibility of the crying of a child in the womb before the evacuation of the waters.—*Id. Ibid.*

---

*On Injections into the vessels of the Umbilical Cord, in cases of retained Placenta.*—Dr. De Berghes has had several opportunities of witnessing the advantageous effects of injections of cold water into the veins of the navel-string, where the placenta is slow in being delivered. He relates four cases which fell under his own immediate notice. In all, the females had passed their fortieth year; they were strong, although lean; and in all of them the mammæ were but little developed. The first case is that of a woman who had borne eight children, and where three hours after delivery, the placenta was still retained. The after-pains were few and feeble, the abdomen timid, the discharge of blood trifling, and the os uteri sufficiently patent. By cutting off a small portion of the navel-string, it was easy to find an umbilical vessel, and to introduce into it the point of a common clyster syringe, with which a pint of cold water was injected. A sensation of chilliness and an increase of the after-pains were produced. A ligature was then applied to the navel-string, and the hand introduced into the womb, where the placenta was found every where adherent. The volume of the placenta, in spite of the injected water, was not large; and the fact of its being injected, and with cold water, very much facilitated its delivery. The second case was somewhat similar, except that the placenta was but partially adherent.

In the third case, where the injection was made about two hours after the birth of the child, and in the fourth half an hour after the child was born, the placenta followed about eight minutes after the injections had been made, and without any assistance. In all the females the subsequent process was undisturbed, and the author is not aware of any injurious effects resulting from the treatment.—*B. & F. M. R., from Wochenschrift für die gesammte Heilkunde.*

*Case of Extirpation of the Parotid Gland, in the Toronto Hospital, by C. Widmer, Esq. Surgeon to the Forces.*

An elliptical incision having been made in the integuments of the most prominent point of the tumour, its removal was effected without much difficulty, and with little loss of blood, the facility being attributed by the author to the adoption of the method of separating the mass of the lower part upwards. The external jugular vein and external carotid artery being necessarily divided, were immediately secured by ligatures, the latter being tied at both ends. When the removal of the mass had been entirely accomplished, the styloid process, and the transverse process of the atlas were exposed to view. The result of the operation was quite favourable, the wound being entirely healed in six weeks.—*London Medical Gazette.*

*On the employment of Gunpowder as a medicine in various morbid states of the Gastro-Enteric Mucous Membrane.*—Dr. Dick, of Glasgow, has lately prescribed this remedy, and, as he states, with satisfactory results. The derangements, for which he thinks it peculiarly adapted, are morbid secretions of the gastro-mucous membrane, dependent on a sub-inflammatory action, or accompanied by it. In such cases, gunpowder, given in various doses, and with the occasional interposition of ordinary mild laxatives, has proved, in his hands, eminently serviceable. Whether it may be appropriate in a greater variety of cases, or whether it may, if given in larger doses, and for a greater length of time, be found useful as a constitutional alterative, or as a cutaneous drug, he is unable to state. Dr. Dick administered from ten grains upwards, several times a day. He found the use of spirituous liquors, or of pungent condiments, &c., rather contra-indicated during the employment of gunpowder. The gastralgic effects which these produce, when used simultaneously with gunpowder, he ascribes to what he designates the *detergent* effects of that substance on the mucous membrane, which, owing perhaps to its charcoal and nitre, it denudes of its attaching albumino-mucous secretion, clearing, and seemingly *attenuating* that membrane, in some measure.

One advantage of this drug is, that it exists in commerce ready for medical use. The best form of administration is in the dry state. No apprehensions need be entertained of the charcoal producing any unpleasant consequences. In *pica*, and in the chlorotic state, large quantities of it are eaten with impunity; and it is further recommended as a remedy in flatulence.—*Edinb. Med. and Surg. Jour.*

*On the Treatment of Varicocele, by Dr. Fricke, of Hamburg.*—In Varicocele, Fricke's treatment consists in drawing a thread directly through the varicose tumour. This method, he says, acts, at least in the greater number of cases, not by producing obliteration of the veins, but by exciting in them a higher contractile power, and an exudation of plastic lymph; thereby inducing a thickening of their coats, and at the same time a diminution of their caliber.

Two cases are mentioned, illustrative of this practice, in one of which, at the end of four months from the commencement of the treatment, no trace of the varicose swelling remained. In the other, two threads were at first passed through the tumour, and nine days later, another thread. After twenty-four days, it was found necessary to repeat the operation, when two more threads were passed; and again, in about six weeks, another. The enlarged veins did not entirely regain their natural condition, but the tumour was greatly diminished, and the testicle, which had exhibited symptoms of incipient atrophy, manifestly increased in size.

*British and Foreign Med. Review.*

*Cauterization of the Eye for Amaurosis.*—In certain cases of amaurosis, particularly in those where an indication exists for acting on the branches of the fifth pair of nerves, M. Serre, of Montpellier, says he has derived very great advantage from cauterizing the surface of the globe with solid nitrate of silver.

*London Lancet from Bul. Therap.*

*On the Treatment of Erysipelas by raw Cotton.*—A number of the Journal des Connaissances Medico-Chirurgicales for last year, contains a recommendation of this article by M. Reynaud, as an excellent application in erysipelas. Its efficacy in burns is well known, and the analogy which exists between inflammation of the skin from this cause, and erysipelas, would seem to suggest its employment in the latter affection. M. Reynaud states that in erysipelas, as in burns, the cotton calms pain as it were by a charm; a mild and moist warmth takes the place of the itching, the formication, the sharp and biting heat which so much increase the pain; the swelling is gradually diminished, the redness disappears, the skin becomes flaccid and wrinkled, and, without becoming covered with those furfuraceous scales, which characterize the termination of erysipelas, and which sometimes continue during a long period. The general excitement and fever disappear with the local phenomena; and the cotton, it is stated, is fitted for all forms of erysipelas, wherever situated, and whether idiopathic or traumatic. It is to be well carded, free from foreign substances, and is to be extended some inches beyond the limits of the inflammation.

